# Vitamin B_1_ and B_12_ Uptake and Cycling by Plankton Communities in Coastal Ecosystems

**DOI:** 10.3389/fmicb.2012.00363

**Published:** 2012-10-12

**Authors:** Florian Koch, Theresa K. Hattenrath-Lehmann, Jennifer A. Goleski, Sergio Sañudo-Wilhelmy, Nicholas S. Fisher, Christopher J. Gobler

**Affiliations:** ^1^School of Marine and Atmospheric Sciences, Stony Brook UniversitySouthampton, NY, USA; ^2^Department of Biological Sciences, University of Southern CaliforniaLos Angeles, CA, USA; ^3^Department of Earth Sciences, University of Southern CaliforniaLos Angeles, CA, USA

**Keywords:** phytoplankton dynamics, heterotrophic bacteria, vitamins, B_12_ limitation, co-limitation, vitamin to carbon ratio

## Abstract

While vitamin B_12_ has recently been shown to co-limit the growth of coastal phytoplankton assemblages, the cycling of B-vitamins in coastal ecosystems is poorly understood as planktonic uptake rates of vitamins B_1_ and B_12_ have never been quantified in tandem in any aquatic ecosystem. The goal of this study was to establish the relationships between plankton community composition, carbon fixation, and B-vitamin assimilation in two contrasting estuarine systems. We show that, although B-vitamin concentrations were low (pM), vitamin concentrations and uptake rates were higher within a more eutrophic estuary and that vitamin B_12_ uptake rates were significantly correlated with rates of primary production. Eutrophic sites hosted larger bacterial and picoplankton abundances with larger carbon normalized vitamin uptake rates. Although the >2 μm phytoplankton biomass was often dominated by groups with a high incidence of vitamin auxotrophy (dinoflagellates and diatoms), picoplankton (<2 μm) were always responsible for the majority of B_12_-vitamin uptake. Multiple lines of evidence suggest that heterotrophic bacteria were the primary users of vitamins among the picoplankton during this study. Nutrient/vitamin amendment experiments demonstrated that, in the Summer and Fall, vitamin B_12_ occasionally limited or co-limited the accumulation of phytoplankton biomass together with nitrogen. Combined with prior studies, these findings suggest that picoplankton are the primary producers and users of B-vitamins in some coastal ecosystems and that rapid uptake of B-vitamins by heterotrophic bacteria may sometimes deprive larger phytoplankton of these micronutrients and thus influence phytoplankton species succession.

## Introduction

Coastal marine ecosystems are amongst the most ecologically and economically productive areas on the planet, providing an estimated US$14 trillion, or about 43% of the global total, worth in ecosystem goods and services, annually (Costanza et al., 1997). While coastal areas comprise only 8% of the world’s ocean surface they account for over 28% of the annual ocean primary production (Holligan and de Boois, 1993). In a manner paralleling global trends, nearly 75% of the US population lives within 75 km of the coastline, making these regions subject to a suite of anthropogenic influences including intense nutrient loading (de Jonge et al., 2002; Valiela, 2006) which in turn can lead to ecological perturbations such as harmful algal blooms and hypoxia (Cloern, 2001; Heisler et al., 2008). Coastal zone management efforts typically focus on minimizing total nitrogen input since primary production in most coastal marine systems are typically nitrogen-limited (Nixon, 1995). While the absolute magnitude of nitrogen entering coastal zones often controls the amount of phytoplankton biomass, the availability, and/or type (e.g., inorganic vs. organic) of nutrients can also influence the algal community composition of coastal environments (Smayda, 1997; Koch and Gobler, 2009). For example discharge from salt marsh ditches, rich in dissolved organic N, elicited a community shift in the adjacent estuarine plankton community favoring dinoflagellates over cryptophytes and cyanobacteria, likely attributable to the mixotrophic tendencies of many dinoflagellates (Taylor, 1987; Anderson et al., 2008; Burkholder et al., 2008). B-vitamins such as thiamin (B_1_), biotin (B_7_), and cobalamin (B_12_) are important cofactors in a number of cellular processes such as the biosynthesis of methionine (B_12_), the decarboxylation of pyruvic acid (B_1_), and fatty acid synthesis (B_7_). While vitamins have long been implicated as growth regulators for microalgae (Droop, 1955; Provasoli and Carlucci, 1974) their ecological importance has received little attention since early surveys (Vishniac and Riley, 1961; Menzel and Spaeth, 1962) and laboratory experiments (Droop, 1968) suggested that ambient concentration were sufficient to satisfy micro-algal demands (Swift, 1980). Newly developed methods to directly measure B-vitamins in seawater (Okbamichael and Sanudo-Wilhelmy, 2004, 2005), have facilitated surveys in several open ocean and coastal ecosystems and have revealed that vitamin concentrations are low, ranging from <0.1–40 to <0.1–100 pmol L^−1^ (Gobler et al., 2007; Panzeca et al., 2008, 2009; Koch et al., 2011) for B_12_ and B_1_, respectively.

Surveys of the literature as well as novel laboratory experiments indicate that over half of all phytoplankton species surveyed have an obligate requirement for an exogenous supply of one or more of the B-vitamins with B_12_ being required by the largest number of algae (Croft et al., 2005; Tang et al., 2010). Vitamin enrichment studies in coastal (Sanudo-Wilhelmy et al., 2006; Gobler et al., 2007) and open ocean environments (Panzeca et al., 2006; Bertrand et al., 2007; Koch et al., 2011) have shown that B-vitamins can co-limit phytoplankton biomass along with a primary limiting nutrient (i.e., nitrogen or iron). Vitamin availability can also shape coastal phytoplankton community structure. Many have argued that B_12_ concentrations might influence the dynamics and composition of the spring bloom (Carlucci and Bowes, 1970; Swift, 1980) since the majority of centric diatom species which comprise the spring bloom are vitamin auxotrophs (Droop, 1955; Guillard and Ryther, 1962). Recently, Koch et al. (2011) demonstrated that vitamin B_12_ concentrations strongly influence algal communities in the coastal Gulf of Alaska, with high concentrations favoring dinoflagellates over diatoms, affirming that B-vitamins can indeed play an important ecological role in plankton succession. Despite this renewed interest in B-vitamins, very little is known regarding how the trophic state of coastal ecosystems influences the cycling of vitamins and only one study has investigated B-vitamin concentrations and uptake rates by plankton communities in an aquatic ecosystem (Koch et al., 2011).

The goal of this study was to elucidate the relationships between phytoplankton community composition, carbon fixation, and B-vitamin (B_1_ and B_12_) assimilation by coastal plankton assemblages. This field study was performed over 2 years in two contrasting, coastal, marine systems: a hypereutrophic and a mesotrophic estuary. In support of the primary objectives of this study, nutrient, and vitamin amendment experiments were performed to explore the extent to which the availability of vitamins affected phytoplankton biomass and community assemblages.

## Materials and Methods

### Study sites

**Figure 1:**
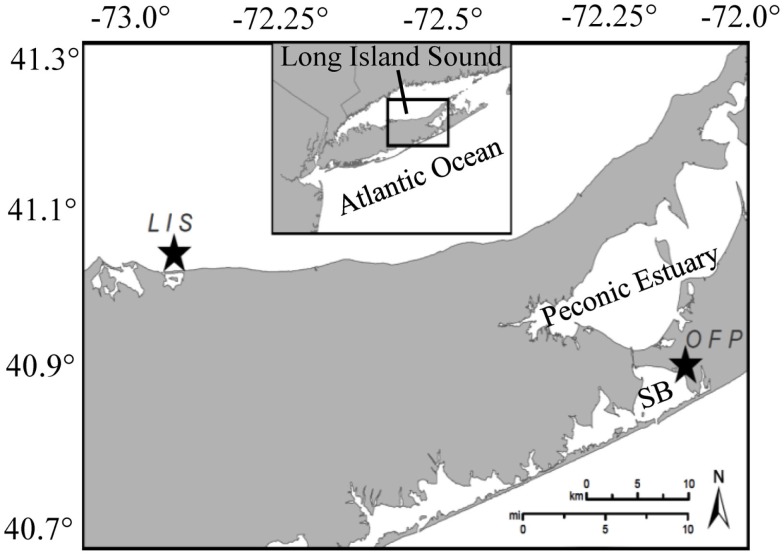
**A map of Long Island, NY showing the two study sites (stars)**. OFP denotes Old Fort Pond, a hypereutrophic systems which exchanges tidally with the Atlantic Ocean through Shinnecock Bay (SB). The second study site, Long Island Sound (LIS), was sampled at the incoming tide from a dock at the Mount Sinai Harbor entrance and represents a mesoeutrophic system.

Two estuaries were investigated during this study and were sampled on 18 occasions on a bi-weekly to monthly basis from March 2007 until November 2008 to capture a complete annual cycle of plankton and vitamin dynamics. Water was collected from the mesotrophic portion of Long Island Sound (LIS) near Mount Sinai Harbor (40.97°N, 73.04°W; Figure 1) and a hypereutrophic, tidal tributary on eastern Shinnecock Bay, Old Fort Pond (OFP, 40.87°N, 72.45°W, Figure 1). LIS is a large, urban estuary bordered on the western end by New York City and in the east exchanges with the North Atlantic Ocean, thus displaying a strong east-west eutrophication gradient (Gobler et al., 2006). Our sampling location was located within the eastern, mesotrophic half of this estuary. OFP exchanges tidally with Shinnecock Bay and is a shallow (<2 m), well-mixed, hypereutrophic body of water which, during the summer months, experiences dense algal blooms that create a large demand for micro- and macro-nutrients and may be influenced by the availability of vitamins (Gobler et al., 2007). This inland, hypereutrophic tributary with high levels of mixed algal biomass (up to 50 μg Chl *a* L^−1^; Gobler et al., 2007) contrasts with the LIS location which typically displays lower levels of algal biomass (mesotrophic; ∼3 μg Chl *a* L^−1^; Gibson et al., 2000).

### Chemical analysis

Water for nutrient analysis was filtered through pre-combusted GF/F filters. Concentrations of nitrate, nitrite, ammonium, and phosphate were determined in duplicate by standard spectrophotometric methods (Jones, 1984; Parsons et al., 1984). Total dissolved nitrogen and phosphorus (TDN, TDP) were analyzed in duplicate by persulfate oxidation techniques (Valderrama, 1981) and dissolved organic nitrogen and phosphorus (DON and DOP) calculated by subtracting levels of nitrate, nitrite and ammonium, or orthophosphate from concentrations of TDN and TDP, respectively. Full recoveries (mean ± 1 SD) of samples spiked with SPEX Certi-Prep^INC^ standard reference material for TDN, TDP, nitrate, nitrite and ammonium, and orthophosphate were obtained at environmentally representative concentrations. Vitamin samples were collected and analyzed according to Okbamichael and Sanudo-Wilhelmy (2004, 2005). Briefly, water was filtered through 0.2 μm capsule filters (GE Osmonics, DCP0200006) into 1 L LDPE bottles and stored frozen and in the dark. The samples were then acidified and concentrated at 1 mL min^−1^ onto columns containing 17% High Capacity C_18_ (Varian, HF BONDESIL), stored frozen, and analyzed via reverse phase HPLC.

### Characterization of the plankton community

Several approaches were utilized to characterize resident plankton communities. Size fractionated chlorophyll *a* (Chl *a*) samples were collected by filtering triplicate samples onto 0.2 and 2 μm polycarbonate filters. These filters were stored frozen until analysis via standard fluorometric methods (Welschmeyer, 1994). Whole seawater was also preserved in 5% Lugols iodine solution for enumeration of plankton (>5 μm) under an inverted microscope. Organisms were identified to the lowest taxonomic level possible and were generally grouped as diatoms, dinoflagellates, ciliates, and autotrophic nanoplankton. A minimum of 200 organisms or 100 grids were be counted per sample (Omori and Ikeda, 1984). Whole water samples were preserved with 10% buffered formalin (0.5% v/v final) and analyzed flow cytometrically to assess picoplankton densities (Olson et al., 1991). Abundance of heterotrophic bacteria (stained with SYBR Green I; Jochem, 2001), phycoerythrin-containing picocyanobacteria, and photosynthetic picoeukaryotes were quantified using a FACScan (BD^®^) flow cytometer using fluorescence patterns and particle size from side angle light scatter (Olson et al., 1991).

### Primary production and vitamin uptake

A ^57^Co-labeled vitamin B_12_ from MP-Biomedicals (specific activity 7.84 MBq μg^−1^; half-life 272 days) and a ^3^H-labeled B_1_ (specific activity 0.37 MBq mmol^−1^; half-life 12.3 years) were used to measure planktonic uptake rates of these vitamins largely following the methods described by Koch et al. (2011). While vitamin B_12_ assimilation was measured throughout this study, vitamin B_1_ assimilation was measured for the second half of this study only (3/7/08 to 11/4/08). Since preliminary studies with these tracers revealed linear uptake rates by multiple types of coastal marine plankton assemblages over 24 h without displaying a diel pattern, incubations were carried out for 1 day. Measured uptake rates never depleted more than 15 ± 6.3 and 7.7 ± 2.2% of vitamin B_1_ and B_12_ standing stocks during these 24 h incubations. Trace amounts (0.5 pM B_12_, 1.48 kBq) of ^57^Co-cyanocobalamin and ^3^H-thiamine (2 pM, 2.22 kBq) were added to separate sets of triplicate, 300 mL polycarbonate bottles. To assess abiotic binding of radiolabeled vitamin B_1_ and B_12_ to particles and, thus, establish a “blank,” several approaches were explored including incubations of natural plankton communities from LIS for 24 h in the dark at 1°C, and with the addition of mercuric chloride and glutaraldehyde at final concentrations of 1%. All of these approaches exhibited similarly low levels of abiotic binding and 1% glutaraldehyde was ultimately chosen as a “killed control” method with one such bottle being spiked with tracer and incubated along the “live” bottles during all experiments. The background activity for detection of ^57^Co was generally between 20 and 40 counts per minute while samples ranged from 80 to 1500 counts per minute.

To determine primary productivity rates, 0.37 MBq of ^14^C-bicarbonate (MP-Biomedicals©, specific activity 2035 MBq mmol^−1^) was added to triplicate bottles according to Joint Global Ocean Flux Study (JGOFS) protocols (1994). All bottles were incubated in an incubator set to mimic ambient light and temperature conditions. Incubations were terminated after 24 h by filtering up to 100 mL from both live and dead bottles onto 0.2 and 2 μm pore size polycarbonate filters, allowing for the determination of size fractionated uptake of the tracers. At the beginning and end of the incubation, a small aliquot of each bottle (250 μL for ^14^C and 1 mL for ^3^H and ^57^Co) was removed to quantify total activity. The ^57^Co containing experimental filters and total activities were analyzed on a LKB Wallac 1282 Compugamma gamma counter equipped with a NaI(Tl) well detector, while the ^14^C and ^3^H samples were measured with a scintillation counter (PackardCarb2100TR). Uptake rates of vitamins B_1_ and B_12_ were calculated by using the equation: [(*A*_f_ − *A*_D_/*A*_tot_) × (vitamin)]/*t* where *A*_f_ is the activity on the live filters, *A*_D_ is the activity on the dead (“killed control”) filters, *A*_tot_ is the total activity added, (vitamin) is the ambient B_1_ or B_12_ concentration and *t* equals the length of the incubation in days. Similarly, uptake of ^14^C-bicarbonate was determined according to the JGOFS (1994) protocol.

Using cell counts obtained via flow cytometry, size fractionated Chl *a* concentrations, estimated carbon contents of heterotrophic bacteria and cyanobacteria and previously published C: Chl *a* ratios, carbon-specific vitamin uptake rates were calculated for both size classes and systems. For the >2 μm plankton, a carbon: Chl *a* ratio of 60 obtained from estuaries including LIS was used (Lorenzen, 1968; Riemann et al., 1989; Boissonneault-Cellineri et al., 2001) while previously published values of average carbon contents of 20 Fg cell^−1^ for heterotrophic bacteria (Fukuda et al., 1998; Ducklow, 2000), and 200 and 250 Fg cell^−1^ for cyanobacteria and picoeukaryotes (Kana and Glibert, 1987; Liu et al., 1999; Worden et al., 2004), respectively were used for the <2 μm size class.

### Vitamin amendment experiments

On each sampling date, water from both sites was used to conduct nutrient/vitamin amendment experiments. Acid-washed, polycarbonate bottles (1.1 L) were filled and amended in triplicate with either 20 μM nitrate, 100 pM B_12_, or both nutrients while another triplicate set was left unamended as a control treatment. The bottles were then incubated for 48 h in OFP mimicking ambient light and temperature conditions (Gobler et al., 2007). To assess phytoplankton responses, bottles were analyzed for size fractionated Chl *a* (0.2 and 2 μm polycarbonate filters) and net growth rates were estimated for the different size fractions based on changes in pigment concentrations using the formula: (*μ* = ln*B*_final_/*B*_initial_)/incubation time where *B*_final_ is the Chl *a* in bottles at the end of experiments, *B*_initial_ is the Chl *a* in bottles at the beginning of the experiments, and the incubation time is days.

### Data analysis

Relationships between environmental parameters were evaluated by means of a Spearman rank order correlation matrix. *p*-values <0.05 were deemed to be significantly correlated and the correlation coefficient is reported as *R*. For nutrient amendment experiments, differences in growth rates among treatments for each size class of plankton pigments were statistically evaluated using two-way analyses of variance (ANOVA) with N and B_12_ as the main effects. *p* < 0.05 was used to establish significant differences among treatments. To assess seasonal trends, the data was also grouped seasonally into spring (March 21–June 20), summer (June 21–September 20), fall (September 21–December 20), and winter (December 21–March 20).

## Results

### Nutrient and vitamin dynamics

**Figure 2:**
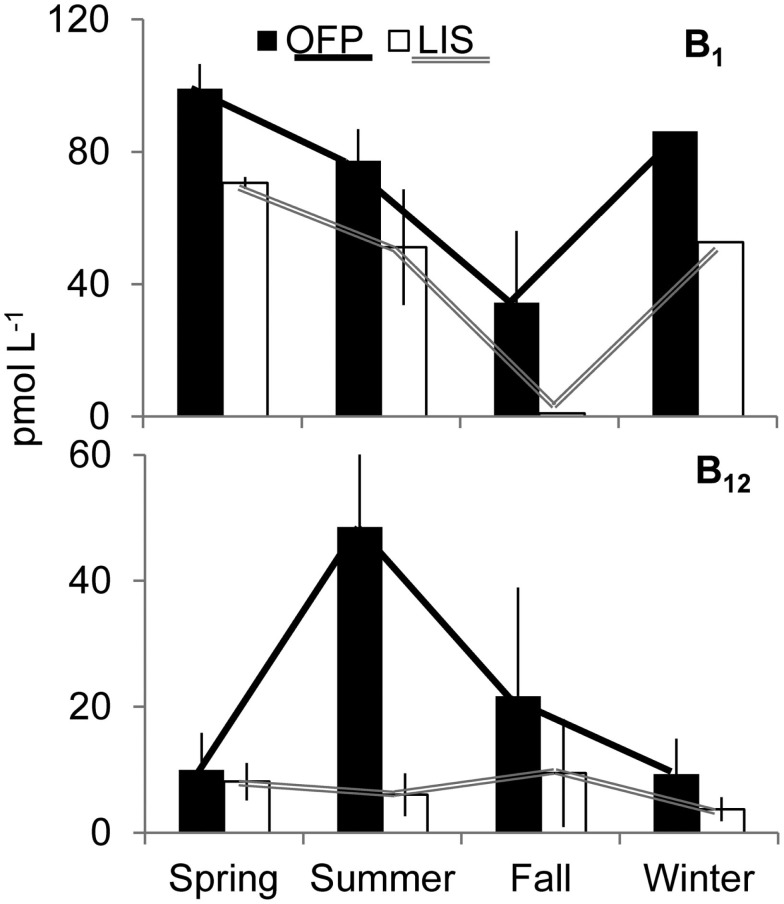
**Seasonally averaged concentrations of B_1_ (top) and B_12_ (bottom) in Old Fort Pond and Long Island Sound, NY**. The solid and hollow lines accentuate the seasonal trend of vitamins in Old Fort Pond and Long Island Sound, respectively. Concentrations are shown in pmol L^−1^.

Dissolved inorganic nutrients and vitamin concentrations in OFP were seasonally dynamic (Figure 2; Table 1). DIN (nitrate + nitrite + ammonium) concentrations varied inversely with water temperature being significantly (*p* < 0.001) higher in the Winter and Spring than in Summer and Fall. In contrast, vitamin B_12_ concentrations (range: <0.1–148 pmol L^−1^; Table 1) were significantly (*p* < 0.05) higher in the Summer and Fall (48.6 ± 32.7 and 21.7 ± 17.3 pmol L^−1^; seasonal average ± SE) than the Spring and Winter (9.95 ± 5.89 and 9.29 ± 4.36 pmol L^−1^, respectively). Concentrations of B_1_ were, on average, higher than vitamin B_12_ (34.9 ± 21.7–99.1 ± 7.46; Table 1) and displayed no clear seasonality (Figure 2). Vitamin B_12_ concentrations were significantly higher in 2008 (55.2 ± 30.7 pmol L^−1^) than 2007 (7.07 ± 1.61 pmol L^−1^; Table 1). In LIS, levels of DIN, DIP, and vitamins were all significantly lower than OFP (Table 1, *p* < 0.05 for all) and generally highest in Winter and Spring. Vitamin B_12_ concentrations in LIS ranged from <0.1–43 pmol L^−1^ and B_1_ concentrations ranged from <0.1 to 99 pmol L^−1^ (Table 1). Unlike OFP, seasonal averages of B_12_ in LIS were higher in Spring and Fall, while levels of B_1_ were highest in the Spring and low (<0.1 pmol L^−1^) in the Fall (Figure 2; Table 1).

**Table 1:** **Physical and chemical characteristics of old fort pond and long island sound over the course of the study**.

	Temperature °C	Salinity	DIN μmol L^−1^	${\text{PO}}_{4}^{-}$ μmol L^−1^	Si(OH)_4_ μmol L^−1^	B_12_ pmol L^−1^	B_1_ pmol L^−1^
**OFP**							
3/26/2007	7.9	29.6	11.2 ± 1.9	0.8 ± 0.1	8.4 ± 1.5	0.3	ND
4/16/2007	9.1	24.4	18.9 ± 1.4	0.2 ± 0.1	12.1 ± 0.0	6.8	ND
4/30/2007	12.5	24.6	21.3 ± 1.6	0.3 ± 0.1	17.2 ± 2.1	7.9	ND
5/21/2007	14.8	28.7	2.2 ± 0.2	0.2 ± 0.0	12.9 ± 0.2	2.7	ND
6/19/2007	21.1	20.5	11.9 ± 2.0	0.1 ± 0.0	43.7 ± 0.1	9.0	ND
7/10/2007	24.6	24.3	0.0 ± 0.2	0.2 ± 0.0	54.8 ± 0.4	3.7	ND
8/8/2007	25.5	26.9	3.5 ± 0.5	0.5 ± 0.0	41.8 ± 0.4	15.5	ND
8/29/2007	ND	ND	1.7 ± 0.3	1.3 ± 0.3	39.1 ± 3.3	17.0	ND
9/26/2007	21.8	28.1	3.5 ± 0.2	0.2 ± 0.0	21.2 ± 6.4	13.0	ND
11/5/2007	11.2	28.5	7.1 ± 2.6	0.3 ± 0.1	10.2 ± 1.3	4.3	ND
12/4/2007	3.9	27.0	3.7 ± 0.2	0.2 ± 0.0	6.0 ± 0.3	1.0	ND
1/8/2008	3.4	25.4	36.2 ± 6.2	0.1 ± 0.0	46.3 ± 4.6	3.7	ND
3/17/2008	5.6	28.2	0.8 ± 0.4	0.1 ± 0.0	3.9 ± 0.5	14.9	86.3
4/14/2008	10.4	27.5	0.8 ± 0.4	0.1 ± 0.0	7.4 ± 0.2	3.1	112.0
5/19/2008	16.2	26.5	29.3 ± 2.5	0.4 ± 0.1	10.9 ± 2.8	38.9	86.1
6/25/2008	23.1	27.8	7.2 ± 0.7	0.9 ± 0.1	30.7 ± 4.9	158.0	79.0
7/30/2008	27.0	25.6	0.8 ± 0.4	0.7 ± 0.1	10.7 ± 0.2	25.1	52.1
9/4/2008	ND	ND	0.4 ± 0.2	0.4 ± 0.1	10.5 ± 0.2	ND	114.6
9/30/2008	20.2	27.2	3.4 ± 0.6	0.4 ± 0.0	21.9 ± 1.1	90.1	68.7
11/4/2008	10.7	28.4	8.9 ± 0.5	0.3 ± 0.0	18.9 ± 0.3	<0.10	0.1
**LIS**							
3/26/2007	3.9	26.0	13.7 ± 0.9	0.3 ± 0.1	3.1 ± 0.6	5.7	ND
4/16/2007	6.2	25.9	13.4 ± 0.9	0.3 ± 0.1	5.4 ± 1.0	0.3	ND
4/30/2007	9.8	24.0	2.8 ± 0.2	0.2 ± 0.0	8.9 ± 3.2	6.0	ND
5/21/2007	11.6	25.0	2.2 ± 0.2	0.5 ± 0.1	13.2 ± 0.6	10.7	ND
6/19/2007	18.2	25.3	2.1 ± 0.6	0.6 ± 0.0	18.0 ± 2.0	2.8	ND
7/10/2007	20.5	26.8	0.8 ± 0.2	0.4 ± 0.1	22.1 ± 0.3	4.0	ND
8/8/2007	23.1	27.1	3.1 ± 0.3	0.9 ± 0.0	35.9 ± 0.4	4.3	ND
8/29/2007	ND	ND	6.4 ± 0.6	2.1 ± 0.3	66.9 ± 3.0	1.9	ND
9/26/2007	21.8	28.1	1.7 ± 0.4	1.3 ± 0.2	26.3 ± 0.6	0.3	ND
11/5/2007	15.1	28.3	12.1 ± 0.9	1.8 ± 0.1	43.3 ± 1.9	2.5	ND
12/4/2007	7.6	28.2	14.1 ± 1.7	1.7 ± 0.1	42.4 ± 5.3	0.9	ND
1/8/2008	4.8	28.2	14.5 ± 3.5	1.9 ± 0.2	50.7 ± 0.9	1.8	ND
3/17/2008	3.7	26.3	1.9 ± 0.2	0.9 ± 0.0	2.4 ± 0.1	5.6	52.7
4/14/2008	6.9	25.7	0.7 ± 0.1	0.6 ± 0.1	2.7 ± 0.2	21.3	73.7
5/19/2008	12.1	24.7	4.1 ± 0.3	0.4 ± 0.0	4.7 ± 0.3	4.7	67.5
6/25/2008	20.0	25.9	1.1 ± 0.5	0.2 ± 0.0	5.3 ± 0.2	0.6	<0.10
7/30/2008	22.1	26.8	2.3 ± 0.4	0.9 ± 0.0	34.5 ± 0.3	27.5	54.8
8/12/2008	21.8	26.7	3.2 ± 0.2	1.0 ± 0.1	35.9 ± 1.2	1.0	98.8
9/30/2008	19.7	25.7	15.8 ± 1.0	1.6 ± 0.0	33.5 ± 12.5	<0.10	<0.10
11/4/2008	11.9	26.7	9.7 ± 0.9	1.7 ± 0.1	38.9 ± 0.5	43.7	<0.10

*Note that B_1_ concentrations were not measured until the second part of the study (3/17/08–11/4/08)*.

### Plankton community succession

**Figure 3:**
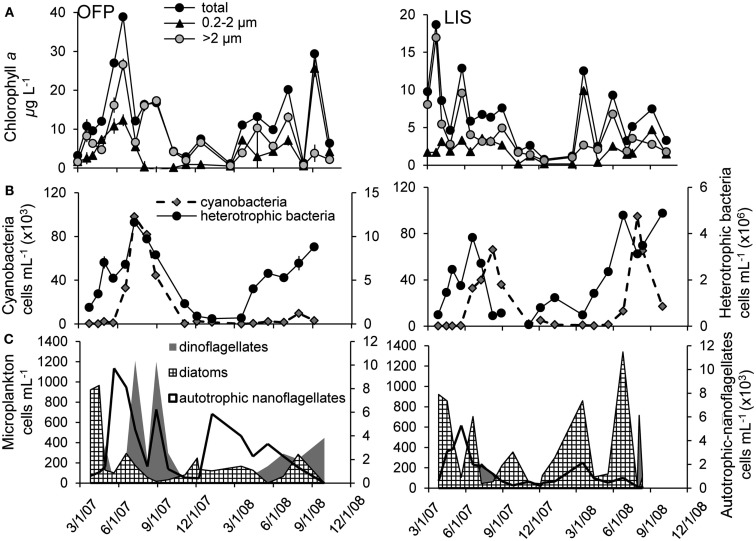
**Planktonic community composition from 2007 to 2008 in Old Fort Pond (left) and Long Island Sound (right), NY**. Size fractionated chlorophyll *a* **(A)** was obtained using varying pore size polycarbonate filters, cyano-, and heterotrophic bacteria concentrations were measured via flow cytometry **(B)** and the nano- and microplankton community was determined via light microscopy **(C)**.

Chl *a* concentrations in OFP varied from 1.12 ± 0.04 to 38.9 ± 0.89 μg L^−1^ (mean = 12.7 ± 2.78; Figure 3A). Size fractionation of Chl *a* revealed a seasonal succession with larger photoautotrophs being most dominant in the Spring, Summer, and Fall and all size classes contributing equally to the algal biomass during Winter (Figure 3A). Heterotrophic bacteria abundances were high, ranging from 0.6 to 11.6 × 10^6^ cells mL^−1^ while densities of picocyanobacteria were lower, 0.11–98.2 × 10^3^ cells mL^−1^ (Figure 3B). Both populations displayed maximal densities during Summer and Fall months with densities of both populations being significantly correlated to water temperature (*p* < 0.01; Figure 3B). The microphytoplankton community in OFP was dominated by autotrophic nanoflagellates and dinoflagellates in the Summer and Fall with diatoms present during the Winter and early Spring only (Figure 3C). In contrast to OFP, the annual mean of plankton biomass in LIS was significantly lower throughout the year (6.55 ± 1.33 vs. 12.7 ± 2.78 μg Chl *a* L^−1^ for OFP), ranging from <1 μg L^−1^ in winter to 18.7 ± 1.79 μg L^−1^ during the spring bloom in 2007 and, with a few exceptions, was dominated by large (>5 μm) diatoms (Figures 3A,C). Heterotrophic bacteria and cyanobacterial abundances in LIS were substantially lower than in OFP with abundances correlating strongly with phytoplankton biomass (Chl *a*, *p* < 0.001) and temperature (*p* = 0.001; Figure 3B).

### Primary production and vitamin uptake

**Figure 4:**
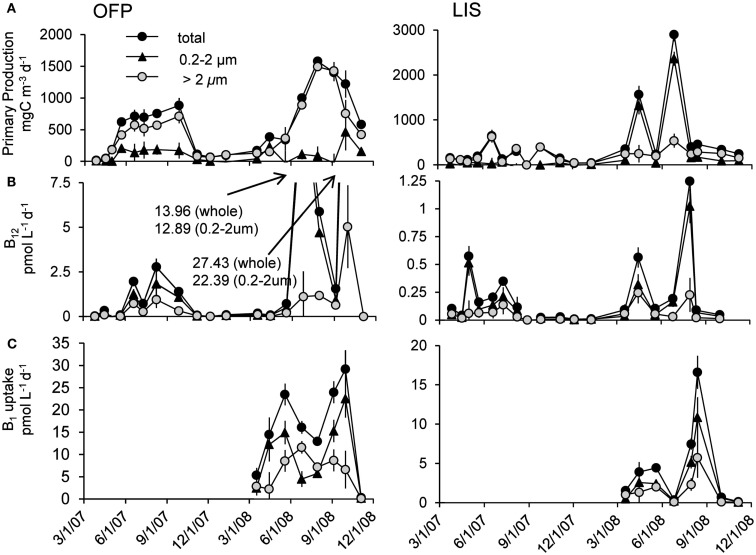
**Time series of primary productivity (A), vitamin B_12_ (B), and B_1_ (C) uptake dynamics in Old Fort Pond (left) and Long Island Sound (right), NY**. Size fractionated primary production and uptake measurements were obtained via the use of polycarbonate filters and are reported in mg C m^−3^ day^−1^ and pmol L^−1^ day^−1^, respectively.

**Figure 5:**
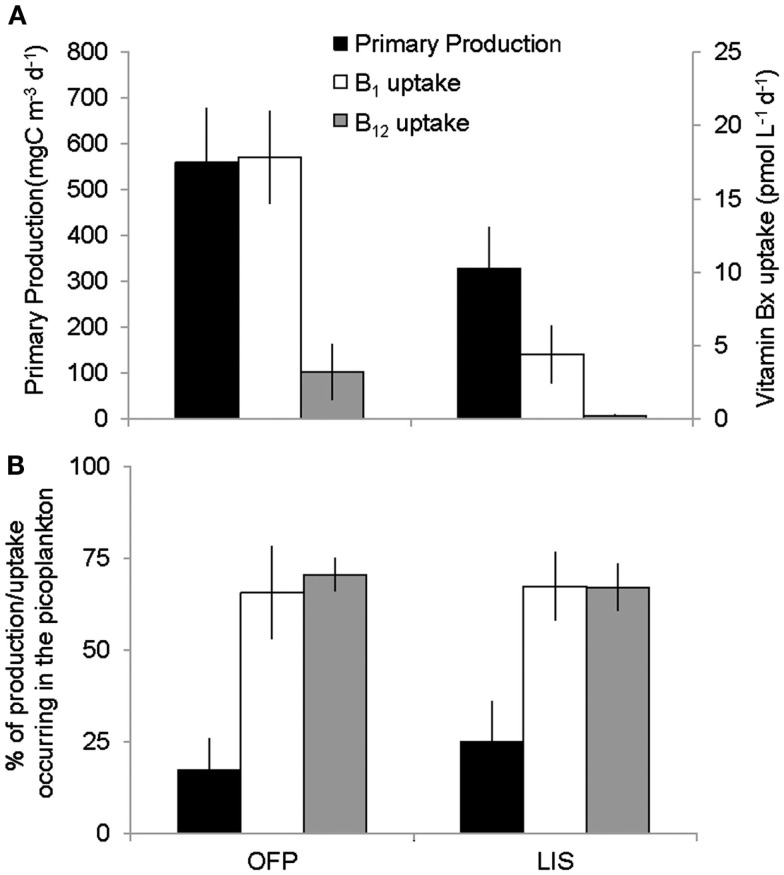
**Seasonal averages of primary production and vitamin B_12_ and B_1_ uptake (A) and the percent of uptake occurring in the picoplankton (0.2–2 μm) size fraction (B) in the two systems studied**. OFP stands for Old Fort Pond while LIS denotes Long Island Sound. Values shown are seasonal means ± SE.

**Figure 6:**
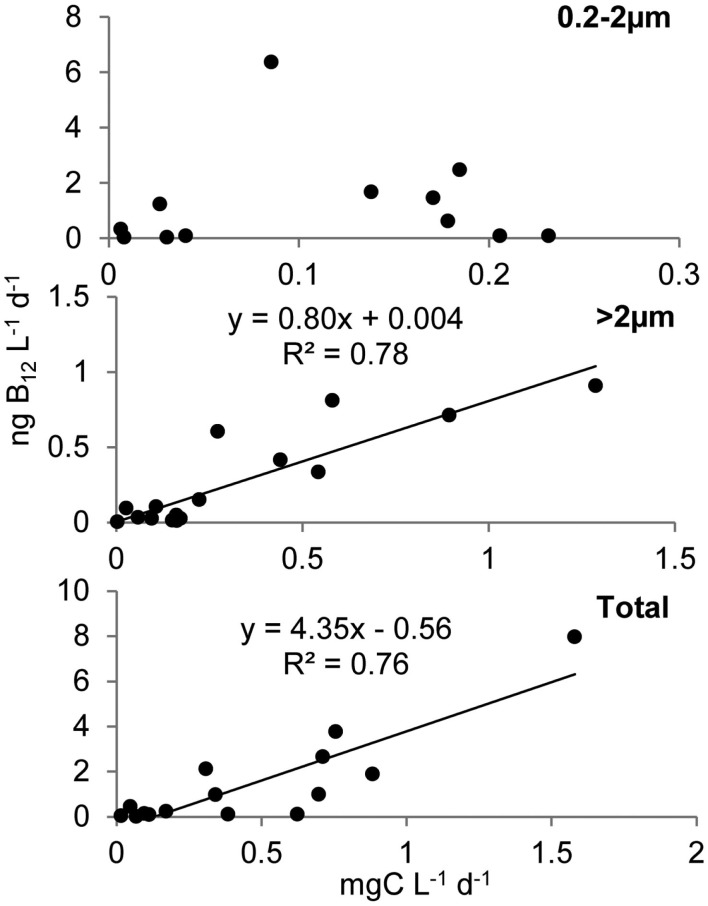
**Relationship between B_12_ and carbon uptake in Old Fort Pond**. Uptake of B_12_ and bicarbonate are reported as ng L^−1^ day^−1^ and mg L^−1^ day^−1^ respectively. Regressions show that in the larger size class (>2 μm), uptake of B_12_ was driven by the photosynthetic organisms in contrast to the <2 μm size class where B_12_ uptake was not correlated to primary production, with heterotrophic bacteria most likely responsible for the majority of vitamin assimilation.

Primary production rates in OFP displayed a strongly seasonal signal with extremely high rates (607 ± 118 mg C m^−3^ day^−1^) in the Summer of both years (Figures 4A and 5). With few exceptions, the majority of this productivity occurred in the >2 μm size fraction (Figure 4A). Vitamin B_12_ uptake followed a similar seasonal trend with uptake rates ranging from <0.1 to 27.4 ± 2.32 pmol L^−1^ day^−1^ and the highest rates observed in Summer and Fall (mean = 3.07 ± 0.57; Figures 4B and 5). In contrast, B_1_ uptake rates, which were higher, displayed little seasonality, ranging from 0.32 ± 0.01 to 29.5 ± 2.45 pmol L^−1^ day^−1^ (mean = 14.4 ± 0.79; Figures 4C and 5). In stark contrast to primary production, picoplankton (0.2–2 μm) were responsible for the majority of vitamin B_1_ and B_12_ uptake in OFP (>65% from Spring-Fall; Figures 4 and 5). While there was no relationship between vitamin uptake and primary production among plankton <2 μm, among larger plankton (>2 μm) these rates were significantly correlated (*r* = 0.88; Figure 6). Notably, the scale of vitamin uptake for the picoplankton was about 10-fold higher than in the >2 μm size fraction.

**Figure 7:**
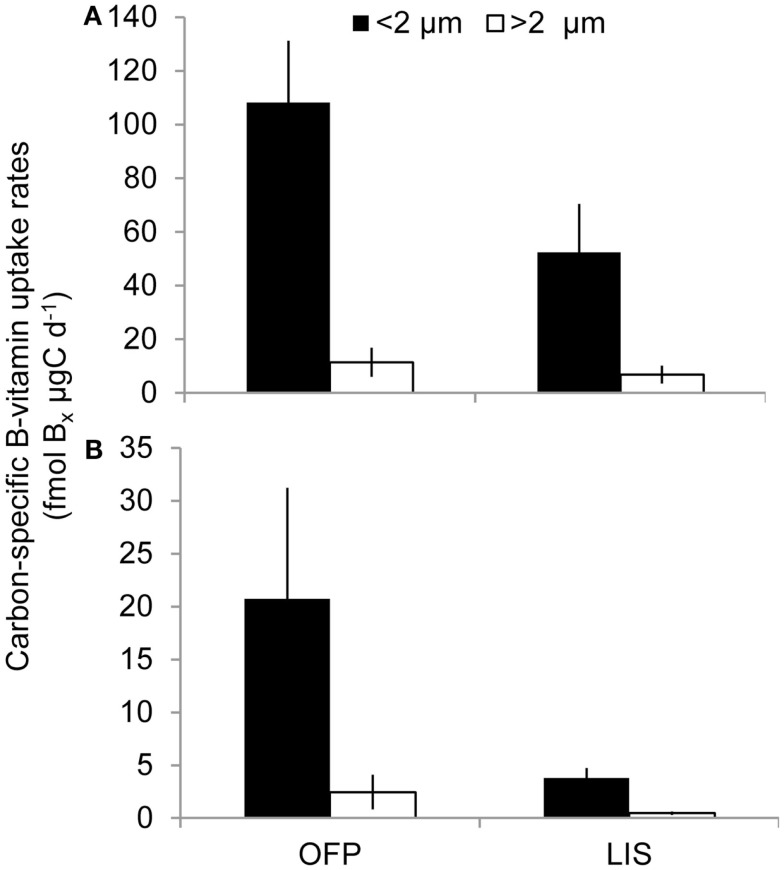
**Average B1 (A) and B12 (B) uptake rates normalized to particulate organic carbon (see Materials and Methods) for each size class and location over the course of the study**. Values are shown as fmol of Bx μg C^−1^ day^−1^, where Bx stands for the corresponding B-vitamin.

Mean primary production rates in LIS (343.4 ± 96.8 mg C m^−3^ day^−1^) were twofold lower than OFP, and peak rates were confined to Spring and Fall of both years (Figures 4A and 5). Like in OFP, larger phytoplankton (>2 μm) accounted for the majority of primary production in LIS (Figure 4). Vitamin B_12_ (0.22 ± 0.08 pmol L^−1^ day^−1^) and B_1_ (4.39 ± 1.96 pmol L^−1^ day^−1^) uptake rates were 10- and 4-fold lower in LIS than OFP, respectively (Figures 4B,C). Like OFP, picoplankton were responsible for the majority of vitamin assimilation in LIS (>55% for all seasons, Figures 4 and 5). Carbon-specific vitamin uptake rates calculated for both size classes and systems revealed striking differences between picoplankton and the >2 μm size class. Specifically, picoplankton utilized an order of magnitude more B_1_ and B_12_ per gram of carbon (Figure 7). In addition, there were 200 and 550% higher vitamin uptake for B_1_ and B_12_ normalized to carbon, respectively in OFP compared to LIS.

### Vitamin amendment experiments

Experiments conducted using water from OFP and LIS revealed that nitrogen frequently stimulated phytoplankton biomass (56% of experiments, Table 2) and generally did so for both large and small phytoplankton (> and <2 μm). While B_12_ additions only occasionally enhanced larger algal biomass (>2 μm; 11% of experiments) it more frequently enhanced the growth rate of the 0.2- to 2-μm size fraction (28% of experiments; Table 2). When added together, nitrogen and B_12_ enhanced total algal biomass more than each individual treatment in 20% of experiments, suggesting a co-limitation of the community by both compounds. The >2 μm size class was more frequently enhanced in this treatment and most of these effects were observed in the Summer and Fall.

**Table 2:** **Responses to nitrogen and vitamin B_12_ amendments by different size classes of phytoplankton collected from OFP and LIS**.

		Total	0.2–2 μm	>2 μm
OFP (*n* = 18)	B_12_	2	4	5
	N	9	5	8
	N + B_12_	2	4	4
LIS (*n* = 18)	B_12_	2	6	4
	N	11	11	11
	N + B_12_	4	2	6
ALL (*n* = 36)	B_12_	4	10	9
	N	20	16	18
	N + B_12_	6	6	9

*Responses for each size fraction were measured via changes in chlorophyll *a* over time and are shown for each sample location. In addition overall effects were examined by looking at all the experiments conducted for each size class (ALL). An effect of the NO_3_ + B_12_ treatments was deemed significant when *p* < 0.05 compared to the NO_3_ and B_12_ only additions. Values represent the number of times a treatment resulted in a positive growth response in that size class*.

## Discussion

Although the potential for B-vitamins to influence the structure and productivity of phytoplankton communities has been recognized for decades, this study is the first to investigate vitamin B_1_ and B_12_ uptake by plankton communities in an aquatic ecosystem. Performing this investigation in two contrasting estuaries (mesotrophic vs. hypereutrophic), this study specifically documented high vitamin concentrations and uptake rates in eutrophic regions that host elevated primary production and heterotrophic bacterial abundances. Although phytoplankton communities were occasionally stimulated by the addition of vitamin B_12_ alone and in tandem with nitrogen, it was the picoplankton community that was responsible for most of the vitamin uptake during this study. The sum of the data collected suggests that within this size class, heterotrophic bacteria were responsible for the majority of the vitamin uptake in both systems and in all seasons.

### Vitamin availability

The two systems studied here displayed vastly different chemical and biological characteristics, with the OFP containing higher nutrient (Table 1), Chl *a*, and vitamin concentrations than LIS (Figures 2–4). While seasonally averaged primary production values did not vary significantly between the sites (Figure 5), OFP had much larger sustained primary productivity rates throughout the Summer and Fall as evidenced by the reduced variability among seasonal averages. Only prokaryotes possess the genes necessary to synthesize vitamin B_12_ (Warren et al., 2002; Rocap et al., 2003; Newton et al., 2010) and the twofold higher heterotrophic bacterial densities in OFP likely resulted in higher rates of *de novo* vitamin synthesis and concentrations. Gobler et al. (2007) found a strong linear relationship between bacterial abundance and B_12_ concentrations in OFP in summer, a time when the highest vitamin concentrations and uptake rates were observed. Cyanobacteria populations can also produce vitamins (Parker, 1977; Palenik et al., 2003; Bonnet et al., 2010) and were also present at higher concentrations in OFP (Figure 3) compared to LIS. Abundances of both groups of picoplankton were strongly correlated with water temperature (*p* < 0.01) a fact which likely contributed to the seasonal peaks in B_12_ observed in OFP during Summer and Fall (Figure 2) but not in LIS where B_12_ concentrations and primary production were less dynamic (Figure 2). For both systems, primary productivity and B_12_ concentrations were strongly correlated (*r* = 0.61; *p* < 0.005) suggesting a tight coupling between photoautotrophs and B_12_ producing prokaryotes. In contrast, vitamin B_1_ was inversely correlated with bacterial biomass (*r* = −0.63, *p* < 0.05) and primary production (*r* = −0.74, *p* < 0.05). Given that vitamin B_1_ requirements of phytoplankton generally exceed those of vitamin B_12_ (Tang et al., 2010) and that vitamin B_1_ uptake rates exceeded those of vitamin B_12_, these inverse correlations may reflect a larger net microbial (phytoplankton and bacteria) uptake of this vitamin.

### Vitamin uptake

B-vitamins were actively utilized by phytoplankton as total uptake rates of B_12_ and total primary production in both systems were highly correlated (*r* = 0.80, *p* < 0.001). Examining this trend among the different size fractions indicated that while primary production and vitamin uptake in the >2 μm size were also positively correlated (*r* = 0.83, *p* < 0.001), this was not true for the picoplankton (<2 μm) despite 10-fold higher vitamin uptake rates in this size class (Figure 6). The strong correlation between vitamin uptake and primary production in the >2 μm size class is consistent with the fact that most phytoplankton are B_12_ auxotrophs (Croft et al., 2005). The absence of such a relationship among picoplankton suggests auxotrophic, heterotrophic bacteria were responsible for the majority of the vitamin use. Peak vitamin uptake rates in OFP exceeded those of LIS by an order of magnitude (Figure 5) and OFP microphytoplankton were dominated by dinoflagellates through much of this study, a group with a high incidence of vitamin B_12_ and B_1_ auxotrophy (91 and 41%, respectively; (Tang et al., 2010). In LIS, phytoplankton communities were generally dominated by diatoms, another group with widespread vitamin B_12_ auxotrophy (Provasoli and Carlucci, 1974; Croft et al., 2005) and diatom abundances in LIS were also positively correlated with vitamin B_12_ uptake by the >2 μm size class (*r* = 0.65, *p* < 0.02).

This study reports the first vitamin B_1_ uptake rates by aquatic plankton communities and reveals important similarities and differences between these rates and those of vitamin B_12_ uptake. Similar to the ambient B_1_ concentrations (Figure 2), uptake rates of that vitamin were an order of magnitude higher than B_12_ uptake rates (Figure 5). Like vitamin B_12_, B_1_ assimilation also occurred primarily in the picoplankton size class (60.0 ± 5.60 and 62.8 ± 5.10% for OFP and LIS respectively; Figure 5) suggesting that bacteria may hold a competitive advantage over larger phytoplankton for access of this micronutrient. Vitamin B_1_ uptake rates in LIS and OFP were generally higher in the Spring, Summer, and Fall, paralleling patterns for vitamin B_1_ concentration (Figure 2), likely due to temperature dependency of microbial metabolisms. Unlike vitamin B_12_, B_1_ uptake rates were not correlated with any plankton group suggesting that multiple plankton groups were important for B_1_ uptake or that groups not definitively documented by this study were important vitamin B_1_ users.

A lack of correlation between primary production and vitamin uptake in the picoplankton (Figure 6), 10- to 100-fold greater heterotrophic bacteria biomass (104 ± 16.0 and 42.2 ± 7.35 μg C L^−1^ for OFP and LIS, respectively) compared to phototrophic picoplankton (3.34 ± 1.48 and 4.44 ± 1.42 μg C L^−1^ for OFP and LIS, respectively), and a strong correlation between heterotrophic bacterial abundances and B_12_ uptake (*p* < 0.008), all suggest that heterotrophic bacteria were the group responsible for the majority of vitamin uptake during this study. Several recently sequenced marine bacterial genomes possess B_12_ dependent enzymes while lacking genes for B_12_ synthesis (Medigue et al., 2005), suggesting that in addition to being B_12_ synthesizers (Warren et al., 2002; Rocap et al., 2003; Newton et al., 2010) marine heterotrophic bacteria might also be important vitamin consumers. Cyanobacteria and picoeukaryote populations in OFP for 2008 were low (<10^4^ cells mL^−1^) when vitamin uptake rates were the highest recorded for the study (Figures 3 and 4), again implicating heterotrophic bacterial community as the most important group for vitamin assimilation. This study highlights the importance of picoplankton in vitamin consumption and suggests that vitamins are primarily assimilated by heterotrophic bacteria, even when larger (>2 μm), eukaryotic cells dominate plankton biomass (>85% of the total POC during spring and summer). As such, previously reported paradoxically low vitamin concentrations in areas of high bacterial activity such as the deeper LIS waters during summer hypoxia (Panzeca et al., 2009) may be due to a large vitamin demand by actively growing heterotrophic bacteria outpacing the vitamin supply or low vitamin production.

### Vitamins alter plankton community composition

In both OFP and LIS, vitamin B_12_ amendments stimulated total Chl *a* production in 12% of experiments (Table 2) while eliciting size structure changes in nearly half of experiments (47%). In OFP, B_12_ limited phytoplankton solely in the Winter and Spring times when vitamin concentrations were lowest (Table 1). In contrast to OFP, phytoplankton in LIS were most limited by vitamins in the summer and fall. The significant correlation between primary production and B_12_ concentrations (*r* = 0.83, *p* < 0.005) as well as between heterotrophic bacterial densities and B_12_ concentrations (*r* = 0.47, *p* = 0.05) suggest that increases in heterotrophic bacteria, fueled by the high primary production, likely led to increased vitamin production and the alleviation of vitamin limitation. As such, the supply of vitamins by bacteria likely influenced vitamin limitation of the phytoplankton in both systems. This conclusion is consistent with Bertrand et al. (2007, 2011) who found vitamins limited phytoplankton communities in coastal regions of the Ross Sea only when bacterial densities were low. As has been previously observed (Gobler et al., 2007; Koch et al., 2011), the addition of B_12_ in conjunction with nitrogen led to increased growth rates over B_12_ or nitrogen alone, a result that may be partly elicited by an increase in B_12_ demand due in the presence of extra nitrogen. This occurred most frequently in the >2 μm size fraction. While uptake rates of vitamin B_12_ were tightly coupled to primary production in this larger size class (Figure 6), the rate of vitamin uptake for that size class was <35% of the total vitamin consumption suggesting that the uptake of the low concentrations of vitamins is dominated by the picoplankton (Figure 4), but that both groups are occasionally limited by the availability of vitamin B_12_.

### A revised notion of B-vitamin cycling

Karl (2002) hypothesized that in marine ecosystems, vitamins are produced by bacteria and utilized by larger phytoplankton and while the importance of B-vitamins in phytoplankton physiology has been well-established, the ecological relevance of these micronutrients in the field has been questioned (Droop, 1961, 1968, 1970, 2007). Although prior culture studies suggested that vitamins are present at high enough concentrations to satisfy algal demand, these studies relied on bioassays to estimate the levels of vitamins in the world’s oceans. Furthermore phytoplankton are often stimulated experimentally by the addition of B-vitamins (Panzeca et al., 2006; Sanudo-Wilhelmy et al., 2006; Bertrand et al., 2007; Gobler et al., 2007; Koch et al., 2011). This paradigm may be best explained by the revelation that, in addition to being the source of B-vitamins (Medigue et al., 2005; Bonnet et al., 2010; Bertrand et al., 2011), prokaryotes are also the main sink for these micronutrients in marine systems (Figures 4 and 5; Koch et al., 2011) and vitamin uptake by these microbes may, on occasion, deprive eukaryotic phytoplankton of a sufficient vitamin supply. The higher vitamin demand by the plankton communities in OFP point to a microbial community adapted to higher vitamin concentrations in this more eutrophic systems with higher ambient vitamin concentrations. The higher carbon normalized B_12_-vitamin demand in OFP may be caused by the denser heterotrophic bacterial populations with a higher percentage of vitamin auxotrophs. Similarly, the dominance of dinoflagellates (Figure 3), a group comprised almost exclusively of auxotrophs (Tang et al., 2010), in OFP likely accounted for the large >2 μm B_12_ demand per unit carbon there (Figure 7). This group was rare in LIS.

Recent work exploring natural ecosystems (Bertrand et al., 2011), cyanobacterial cultures (Bonnet et al., 2010), and prokaryotic genomes (Raux et al., 2000; Palenik et al., 2003; Newton et al., 2010) have highlighted the important role of prokaryotes in ocean vitamin production and consumption. While it is unknown whether specific groups of vitamin producers are also responsible for vitamin assimilation, this would seem counterintuitive since vitamin biosynthesis is complicated and, in the case of B12 utilities numerous genes and enzymatic steps (Raux et al., 2000; Warren et al., 2002). Due to the demanding nature of this process, B-vitamin auxotrophic bacteria may harbor an energetic advantage over vitamin-producing bacteria and seem to hold a kinetic advantage in assimilating picomolar levels of vitamins over larger, vitamin auxotrophic phytoplankton likely due to their larger surface to volume ratio (Raven and Kubler, 2002). In addition, the faster doubling times and higher biomass of heterotrophic bacteria (Giovannoni et al., 2005) likely leads to a much higher vitamin demand compared to larger, slower growing phytoplankton and the development of vitamin depleted surface oceans observed by Sañudo-Wilhelmy et al. (2012). Regardless, we hypothesize that vitamin cycling (uptake and production) occurs primarily within the prokaryotic picoplankton community, a factor which may limit the growth of some eukaryotic phytoplankton cells.

## Conflict of Interest Statement

The authors declare that the research was conducted in the absence of any commercial or financial relationships that could be construed as a potential conflict of interest.
